# Aging with ING: a comparative study of different forms of stress induced premature senescence

**DOI:** 10.18632/oncotarget.5947

**Published:** 2015-10-01

**Authors:** Uma Karthika Rajarajacholan, Karl Riabowol

**Affiliations:** ^1^ Department of Biochemistry and Molecular Biology, University of Calgary, Calgary, Alberta, Canada; ^2^ Department of Oncology, Faculty of Medicine, University of Calgary, Calgary, Alberta, Canada

**Keywords:** senescence, ING1a, epigenetic, Retinoblastoma, p16, Gerotarget

## Abstract

Cell senescence contributes to organismal aging and is induced by telomere erosion and an ensuing DNA damage signal as cells reach the end of their replicative lifespan *in vitro* or *in vivo*. Stresses induced by oncogene or tumor suppressor hyperactivation, oxidative stress, ionizing radiation and other DNA damaging agents result in forms of stress induced premature senescence (SIPS) that show similarities to replicative senescence. Since replicative senescence and SIPS occur over many days and many population doublings of the mass cultures of primary cells used to study senescence, the sequence of events that occur downstream of senescence signaling can be challenging to define. Here we compare a new model of ING1a-induced senescence with several other forms of senescence. The ING1a epigenetic regulator synchronously induces senescence in mass cultures several-fold faster than all other agents, taking 24 and 36 hours to activate the Rb/ p16^INK4a^, but not the p53 tumor suppressor axis to efficiently induce senescence. ING1a induces expression of intersectin 2, a scaffold protein necessary for endocytosis, altering the stoichiometry of endocytosis proteins, subsequently blocking growth factor uptake leading to activation of Rb signaling to block cell growth. ING1a acts as a novel link in the activation of the Rb pathway that can impose senescence in the absence of activating p53-mediated DNA damage signaling, and should prove useful in defining the molecular events contributing to Rb-induced senescence.

## INTRODUCTION

Cells grown *in vitro* offer a homogeneous and manageable system to study biochemical and genetic complexities in an organism. Cells explanted from vertebrates were once thought to grow indefinitely in culture until Hayflick and Moorhead challenged this view using human fibroblasts and confirmed that normal diploid cells explanted from tissues had a limited replicative life span in culture [1]. They observed that normal primary fibroblasts, after undergoing a fixed number of divisions, entered a state of cell cycle arrest, where they remained viable and metabolically active but lost their replicative ability. This phenomenon of replicative exhaustion was termed ‘replicative senescence’ and subsequent studies demonstrated a link between the length of telomeres found at the ends of chromosomes and the replicative life span of cells grown *in vitro* [2, 3]. Progressive loss of telomere DNA of the sequence TTAGGG_n_ after every cell division results in telomere length shortening until telomeres, or a subset of telomeres in the cell reach a critical length that initiates a DNA damage signal culminating in replicative arrest [2]. The limited number of divisions exhibited by explanted primary cells appears to contribute to the limited life span of the donor organisms and thus replicative senescence appears to constitute an informative model to study the molecular events initiating key aspects of organismal aging [4, 5]. Senescent cells show activation of p53 [6], decreased ability to inactivate Rb [7] and the fact that expression of viral oncoproteins that inactivate the p53 and Rb proteins results in immortalization of cells suggested that these two tumor suppressor axes play crucial roles in enforcing cellular senescence [8]. Along with apoptosis, senescence is now considered a major evolutionarily conserved tumor suppressor mechanism that prevents the division of old, damaged or mutant cells. While apoptosis removes the abnormal cells by programmed death, senescence prevents their continued division. Thus, understanding cell senescence would bridge our understanding of pathways governing both cancer and aging [3, 9, 10].

Although telomere length is the molecular clock that regulates the number of times a normal cell can divide, several studies have shown that replication-associated telomere attrition is not the only process that can induce cellular senescence. Cells also show many senescence-associated phenotypes as a consequence of physiological insults. This form of senescence is called stress-induced premature senescence or SIPS, and occurs independently of telomere length. Several intrinsic factors have been identified that cause SIPS in cells grown in culture. These include suboptimal culture media conditions, high oxygen levels, and the absence of cellular microenvironments. SIPS can also be a result of extrinsic factors including oxidative stress (growing cells in oxygen-rich conditions or exposure to hydrogen peroxide and t-butyl-hydroperoxide), DNA damaging agents (ionizing radiation and chemotherapeutic agents), oncogene hyperactivation or over-expression (Ras, BRAF) or abnormal tumor suppressor levels (p16^INK4a^, p53) [9]. The number of agents found that can induce SIPS in cells is growing with time and most of them function through DNA damage response pathways. These observations suggest that like replicative senescence, SIPS also functions as a protective mechanism to prevent replication of damaged cells that harbor mutations or other abnormalities that could potentially lead to cancer.

The epigenome is altered during cell aging and many studies indicate that it contributes to the aging process itself [11]. Consistent with this, we previously found that expression of the ING1a epigenetic regulator increased several-fold during replicative senescence. ING1a is one isoform of ING1 that encodes two major isoforms, p33ING1b that induces apoptosis when overexpressed and p47ING1a that induces senescence. ING1 is the founding member of a family of epigenetic regulators (ING1-5) with conserved plant homeodomains (PHDs) that function as readers of the epigenetic code to target both lysine acetylase and lysine deacetylase complexes to chromatin (reviewed in [12,13]). Ectopic expression of ING1a in replication-competent, low passage (“young”) cells induced many phenotypic changes typical of replicative senescence such as increased cell and nuclear size, the appearance of senescence associated heterochromatic foci (SAHF) containing HP1γ, and SA-β−Gal staining, among others [14]. Examination of the transcriptome affected by ING1a indicated a novel mode of senescence induction by dysregulation of endocytosis [15]. This resulted in attenuation of major growth factor signaling including EGFR, Akt and ERK leading to hyper-activation of Rb through both increased Rb levels and the induction of the p16^INK4a^ and p57^KIP2^ CDK inhibitors. We found that expression of ING1a caused cells to become unresponsive to mitogenic stimuli by blocking endocytosis, leading to nutrient deprivation and cell cycle arrest. We also noted that this phenomenon was essentially identical in replicatively senescent cells and knocking down ING1a partially abrogated the loss of response to mitogenic stimulation seen in senescent cells [15]. Given the striking similarity of ING1a-induced, and replicative senescence, we undertook experiments to compare the characteristics of ING1a induced senescence to other forms of SIPS. This study demonstrates that ING1a causes a rapid form of *in vitro* cellular senescence that can better define the temporal relationships between genetic and biochemical pathways and components that are responsible for initiating and enforcing senescence and so can serve to better elucidate key events in cell and by extension, organismal aging.

## RESULTS & DISCUSSION

### ING1a-induced senescence occurs rapidly compared to other forms of SIPS

**Figure 1:**
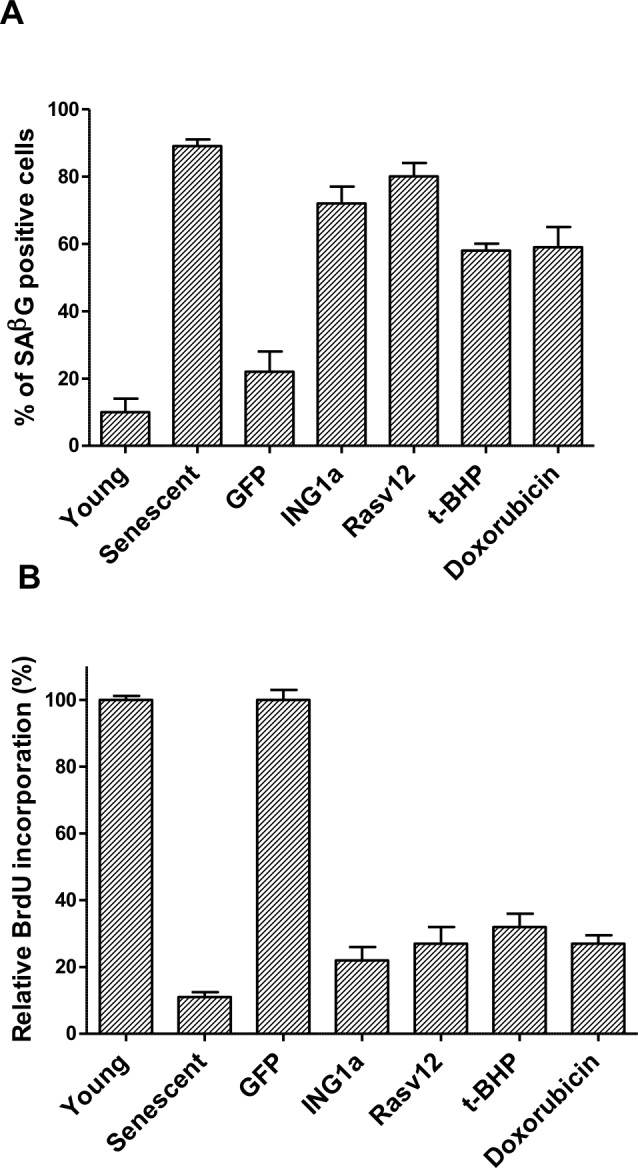

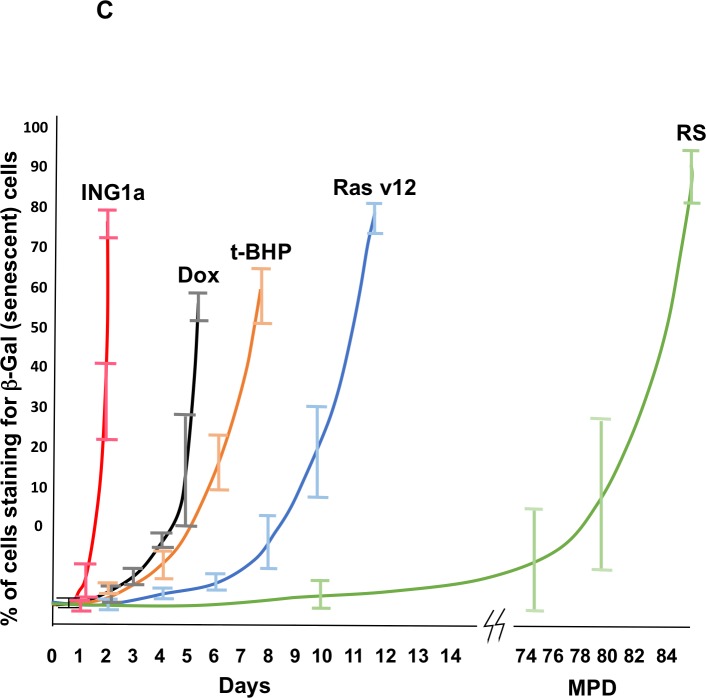
ING1a- induced senescence is a rapid model of RS **A.** percentage of cells staining positive for SA-β-gal staining. Cells were counted after exposure to various agents. SA-β-gal staining was performed in three independent experiments and each time a total of 100-120 cells were counted using a phase-contrast microscope. The values for RS was compared to low passage “young”, proliferation-competent cells and Ad-ING1a and Ad-Rasv12 expressing cells were normalized to the Ad-GFP set. The various treatments were normalized to the young, proliferation-competent set (*n* = 3; *p <* 0.05, student *t* test). **B.** Percentage of cells that incorporated BrdU after the various treatments is presented in the histogram, which are normalized to their respective controls as in Figure 1A (*n* = 4; *p <* 0.05, student *t* test). **C.** Schematic of the time taken for the various agents to induce SIPS as estimated by SA-β-Gal staining. While ING1a, Dox, Ras v12 and t-BHP induces telomere independent senescence in several days, telomere dependent replicative senescence requires several months of passaging before cells reach the end of their replicative life span. MPD stands for mean population doublings. ING1a is the most rapidly acting agent and induces senescent phenotypes within 36-48 hours. After pilot experiments were run, assays were done in triplicate at 12, 24, 36 and 48 hours for ING1a, daily for Dox, every two days for t-BHP and Ras v12 and every 5 days for cells undergoing replicative senescence. For replicative senescence, vials of Hs68 cells frozen at different passages were used, perhaps accounting for relatively larger error bars (showing SD) at higher MPD levels.

We compared ING1a induced senescence to Ras (oncogene-induced), t-butylhydroperoxide [t-BHP] (oxidative stress-induced) and doxorubicin [Dox] (genotoxic stress-induced) forms of SIPS in low passage replication competent (young), normal diploid Hs68 fibroblasts. Hs68 cells grown under standard growth conditions were passaged for several months until their growth slowed to 20% of the rate of young cells at which point most cells in the mass culture were undergoing replicative senescence (RS) and served as a positive control for senescence. Cells treated with the agents noted above were tested periodically for SA-β-gal staining and BrdU incorporation and times were defined for each treatment that resulted in the maximal average proportion of senescent cells being obtained, shown in Figures 1A & 1B. While ras-v12 expressing cells exhibited senescence in 10-12 days, t-BHP treatment induced senescence in 8-9 days and doxorubicin induced senescence in 6-7 days. In contrast, ING1a induced senescence by 36 hours and became maximal by 48 hours. Graphing of the time courses of senescence induction shown in Fig 1C highlights the compressed, and synchronous nature of ING1a-induced senescence compared to the other models of SIPS. As noted previously, this is due to the induction of intersectin 2 (ITSN2) within 24 hours by ING1a binding the ITSN2 promoter, and both p16^INK4a^ and Rb genes being subsequently induced by 36 hours as shown in Supplementary Figure 1.

### ING1a-induced senescence occurs in the absence of activating the p53-p21 axis and strongly correlates with activation of p16^INK4a^ -Rb pathway

**Figure 2:**
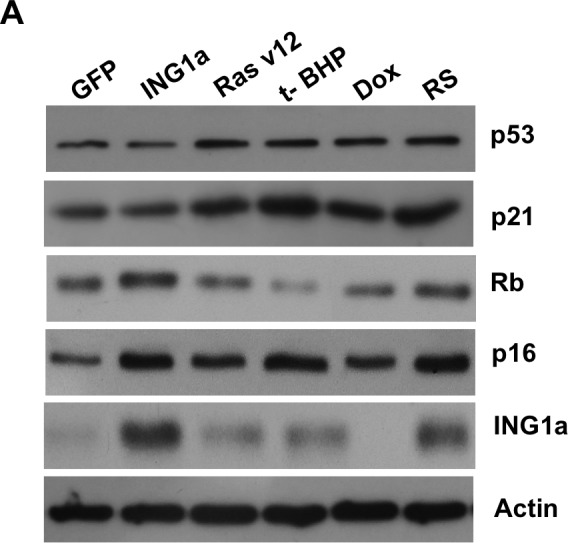

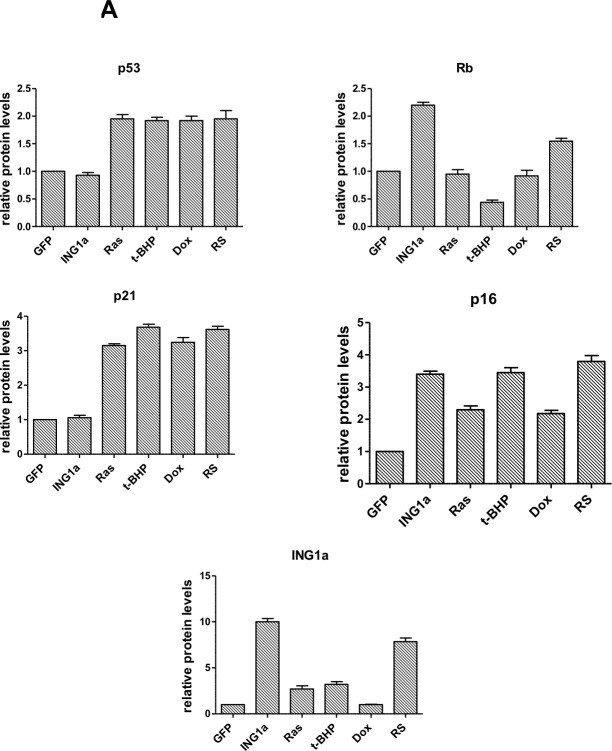

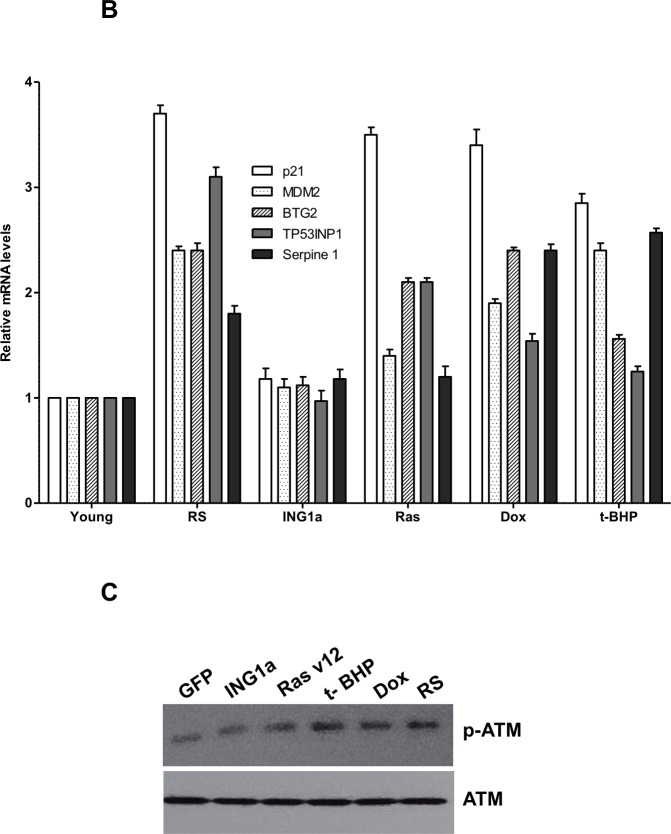

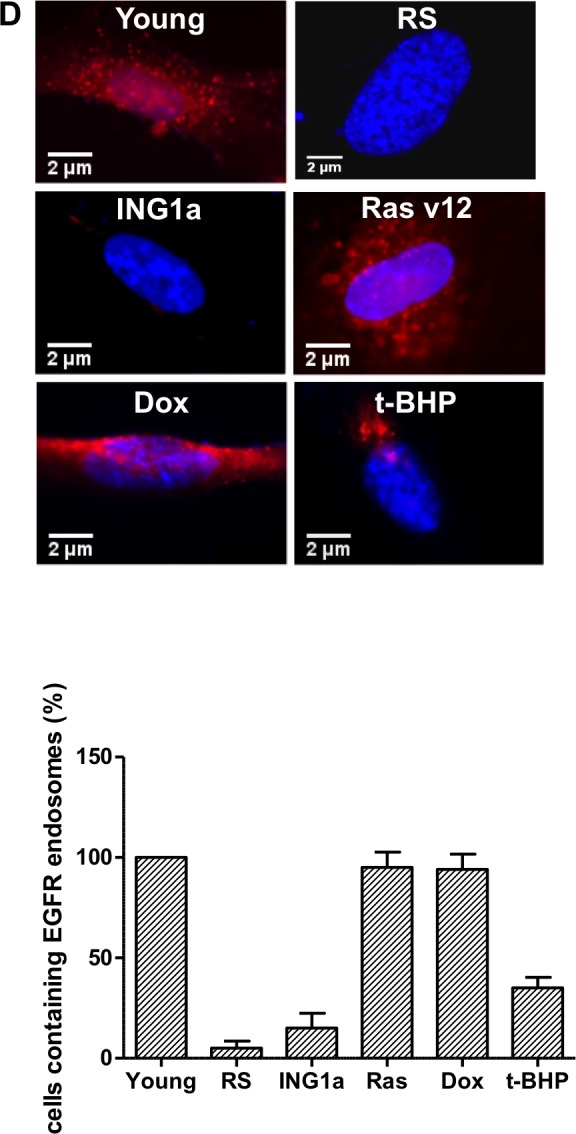
ING1a induced senescence is independent of p53 **A.** Western blots of Hs68 cell lysates that underwent SIPS and RS to check the levels of p53, p21, p16^INK4a^, Rb and ING1a. Actin served as a loading control. The histogram on the lower panel represents the quantification of the results of the western blots from three experimental repeats. **B.** A representative group of p53 targets that are known to function in p53-dependent senescence was analyzed by quantitative real time PCR in each of the SIPS treatments and their levels were normalized to the control set. **C.** p-ATM and total ATM levels were measured by western blotting in the various SIPS induced samples as a marker of DNA damage. **D.** Cells exposed to various SIPS agents were serum starved overnight and stimulated with Texas-Red EGF for 4 minutes to check for internalization of EGF receptor. Percentage of cells possessing EGFR endosomes (marked by the red punta) were counted for every 100 cells and plotted as a graph in the lower panel. The experiment was performed thrice and each time 100 cells were counted. The count on GFP expressing population was taken as 100% and the values were normalized to this population (*n* = 3, *p <* 0.05, student *t* test).

We next wanted to study the effects of the different SIPS-inducing agents on the Rb- p16^INK4a^ and p53-p21 tumour suppressor axes. To do this, lysates from young primary Hs68 cells subjected to different SIPS agents for the times noted above were blotted for expression of the proteins shown in Fig 2A. ING1a levels increased during replicative senescence as noted previously, and also increased slightly after treatment with Ras v12 or t-BHP, but not with Dox. Consistent with ING1a inducing changes similar to those seen in replicative senescence, Rb and p16^INK4a^ levels increased to similar degrees in response to ING1a expression and replicative senescence. Paradoxically, Ras v12, t-BHP and Dox treatment differentially affected p16^INK4a^ and Rb levels, indicating that for the Rb axis, ING1a induced senescence shows the most similarity to replicative senescence. Levels of p16^INK4a^ were induced in all the modes of SIPS, although to varying degrees. We also analyzed a representative set of p53 target genes previously reported to be induced during p53-dependent senescence [16]. Quantitative real time PCR results showed that all the SIPS agents, except ING1a, showed increased transcript levels of the p53 target genes tested, albeit to varying degrees (Figure 2B). ING1a, however, showed minimal or no induction of these transcriptional targets of p53 suggesting that it acts independent of the p53-p21 axis of senescence induction. We next assayed if the activation of p53 and induction of p53 target genes could be a result of persistent DNA damage signaling. To test this, we measured the levels of phospho ATM in the cell lysates of the various SIPS inducing agents and compared them to the control. We found that treatments with Dox, t-BHP and RS had significantly increased levels of p-ATM. While Ras v12 expression had a modest increase in p-ATM levels, ING1a expressing cells did not show any persistence of DNA damage signaling as evidenced by the low levels of p-ATM (Figure 2C). These results confirm that ING1a induced senescence occurs independent of the ATM/DNA damage signaling pathway known to be activated during replicative senescence and the downstream p53-p21 pathway. Consistent with results from our previous report on ING1a blocking growth factor signaling, when cells were examined for their ability to internalize the EGF receptor in response to ligand stimulation, ING1a was nearly as effective as replicative senescence in blocking endocytosis while other forms of SIPS were not (Ras v12, Dox), or were only minimally (t-BHP) effective (Figure 2D).

### SIPS and the formation of senescence associated heterochromatic foci (SAHF)

**Figure 3:**
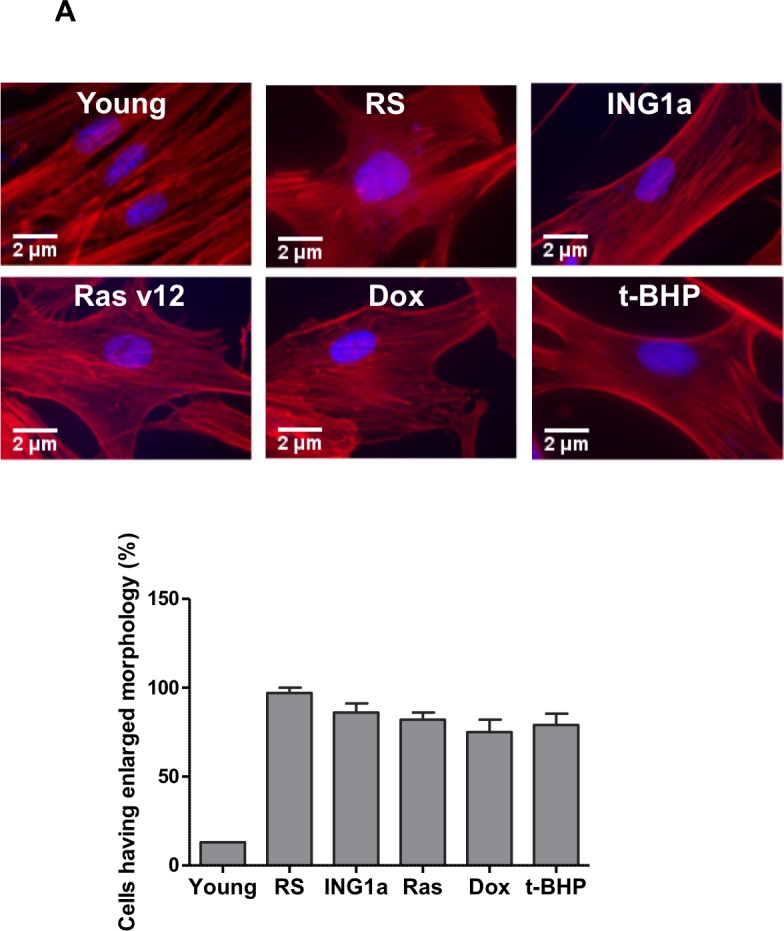

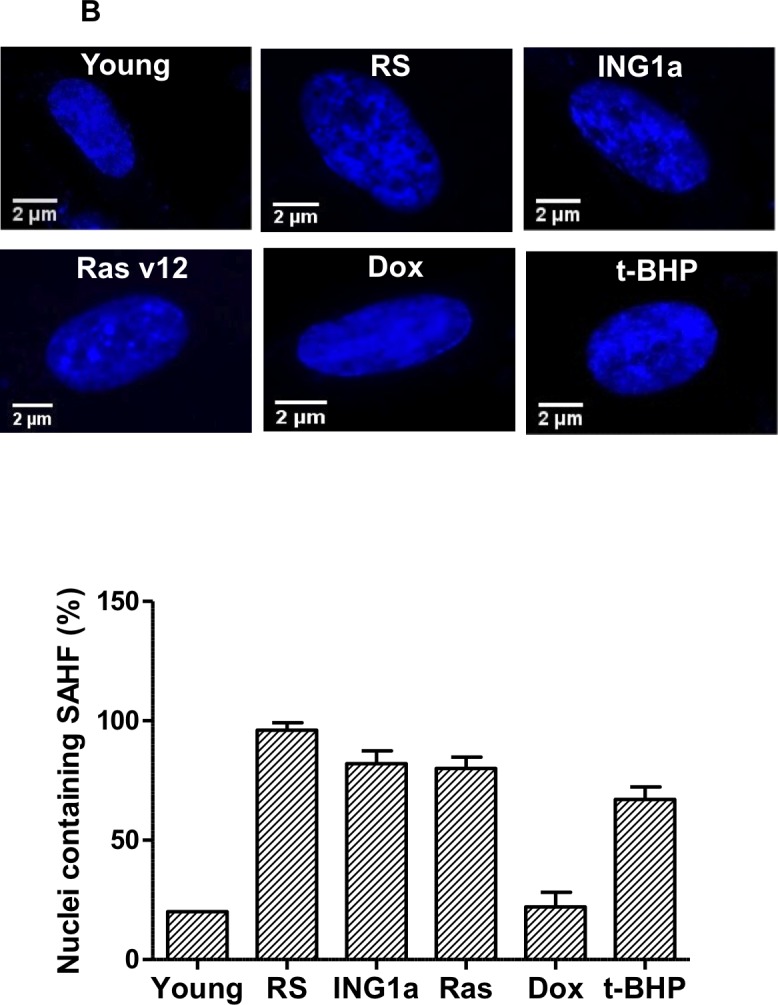

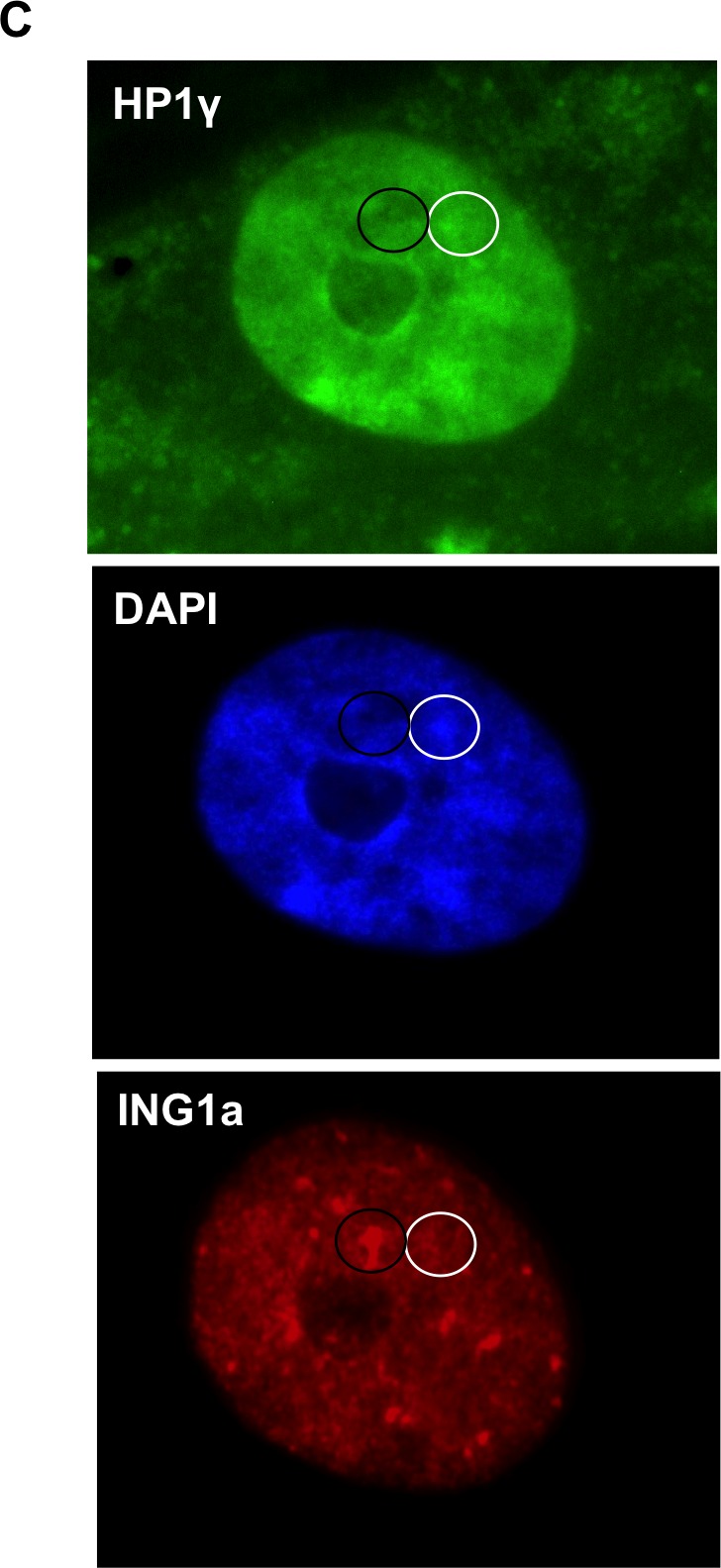
Comparison of senescence markers induced by SIPS **A.** Phalloidin-TRITC staining of Hs68 cells subjected to different SIPS-inducing treatments. The population of cells possessing a flat, enlarged morphology was quantified in the graph in the lower panel. **B.** Cells exposed to the various treatments were fixed and stained with DAPI to assess nuclear morphology and the presence of SAHF. The graph in the lower panel estimates the number of nuclei possessing ≥ 10 DAPI dense regions. 100 nuclei were counted in each of the three replicates carried out (*p < 0.04*). **C.** Cells transfected with ING1a was assayed for ING1a localization and the formation of SAHF. We tested the localization of ING1a (stained red) with HP1γ (green) containing foci. HP1γ is a marker for SAHF.

A common feature of cell senescence is increased cell size and the formation of SAHF containing Rb [17]. Since ING1a was unique in its ability to up-regulate Rb and p16^INK4a^ to levels seen during replicative senescence, we examined the effects of ING1a compared to other inducers of SIPS on these parameters. As shown in Figure 3A, all treatments caused cells to enlarge, flatten and develop prominent actin filaments, as evidenced by phalloidin staining. In addition, all agents caused nuclei to enlarge and, with the exception of Dox, to develop heterochromatic regions evidenced by dense DAPI staining (Figure 3B). The formation of SAHF was initially reported to require the effects of an intact Rb pathway. This is consistent with the robust activation of Rb and p16^INK4a^ by ING1a and replicative senescence (Figure 2A). Furthermore, all agents that induced ING1a (Figure 2A) also induced SAHF formation (Figure 3B). Although Dox was able to induce senescence as measured by SA-β-gal, it was unable to induce ING1a (Figure 2A) or the formation of SAHF (Figure 3B). To test if ING1a had direct physical involvement in SAHF formation as is the case for Rb, we examined the localization of ING1a. As shown in Figure 3C, ING1a did not clearly co-localize with SAHF, suggesting an upstream role for ING1a in SAHF formation. However, although there was no obvious co-localization seen, we cannot rule out some degree of co-localization of ING1a with heterochromatic foci or HP1γ due to residual diffuse staining seen for ING1a, DNA and HP1γ in nuclei. The precise contribution of ING1a in the nuclear heterochromatinization leading to SAHF formation and gene silencing is yet to be understood, although it is likely to be a consequence of blocked endocytosis as seen in replicative senescence [15].

### ING1a expression induces phenotypic aspects of senescence in other cell types

**Figure 4:**
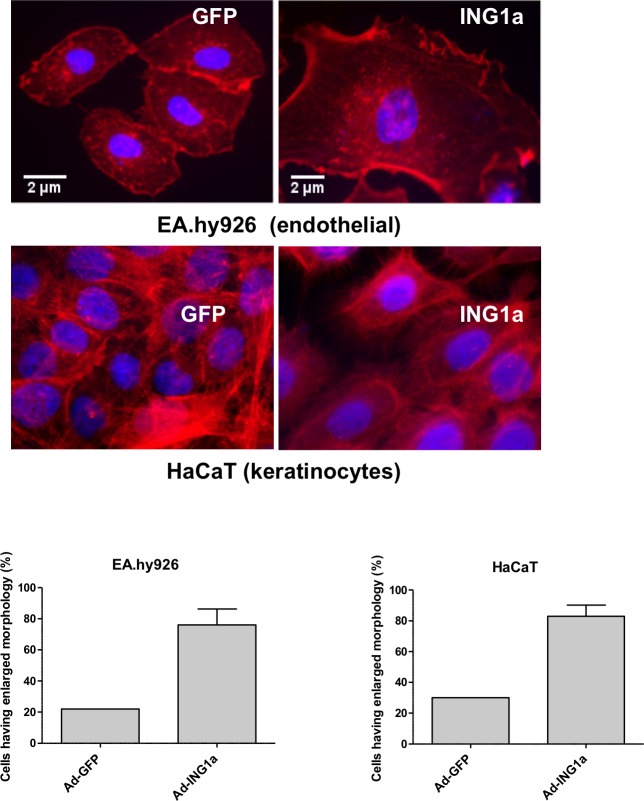
ING1a induces changes to the actin cytoskeleton in primary endothelial cells (top panel) and keratinocytes (bottom panel) The lower panel shows quantification based on counting 200 cells in each of our three independent experimental repeats and is plotted as a percentage of the population of cells showing an enlarged, flat morphology typical of senescent cells.

To test if ING1a could induce features of senescence in other cell types with many characteristics of primary cells, we ectopically expressed ING1a in endothelial cells (EA.hy926) and keratinocytes (HaCaT). We found that ING1a expressing cells showed significant changes in cytoskeletal structure and actin filaments upon phalloidin staining and alterations in nuclear morphology reminiscent of senescence in these cell types (Figure 4). Endothelial cells, and to a lesser extent keratinocytes with elevated levels of ING1a showed increased size, lower density in culture with flattened nuclear morphology, much like fibroblasts overexpressing ING1a. Similar but less dramatic effects of ING1a expression were seen in human umbilical cord endothelial cells and vascular smooth muscle cells (data not shown). It should be noted that although these cells have many characteristics of primary cells, they are immortalized and so cannot be considered “primary” although they have many primary cell characteristics. Nevertheless, they do respond at the level of phenotype, very similarly to primary diploid fibroblasts.

**Figure 5:**
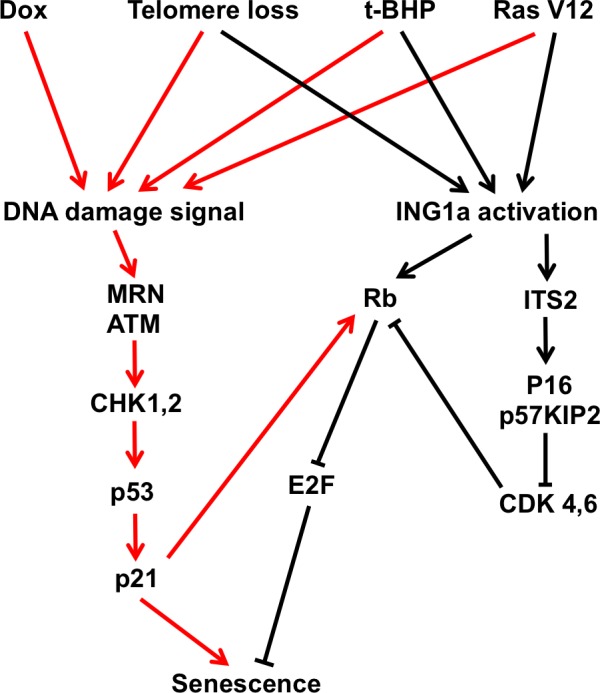
Contributions of the p53 and Rb tumor suppressor axes to cell senescence Telomere attrition, Dox, Ras v12 and t-BHP-induced SIPS trigger a DNA damage signalling cascade including the ATM and CHK kinases, resulting in activation of p53 and senescence induction. In contrast, ING1a primarily functions to reinforce senescence through activation of Rb expression and by inducing the p16^INK4a^ and p57^KIP2^ cyclin dependent kinase inhibitors that normally function to prevent Rb inactivation. Some of the other agents that activate SIPS also partly activate ING1a, supporting the idea of crosstalk occurring between the p53 and RB axes to cause cellular senescence.

Taken together, this and our previous study show that ING1a very rapidly induces senescence through blocking mitogenic stimulation and subsequent activation of the Rb pathway. This likely occurs through the ITSN2-dependent induction of p16^INK4a^ and p57^KIP2^ and ITSN2-independent induction of Rb by ING1a as outlined in the model of ING1a-induced senescence shown in Figure 5. Our preliminary data also suggest that ING1a-induced senescence occurs in the absence of obvious telomere attrition, further supporting the idea that it acts independently of DNA damage signalling. Whether ING1a-induced senescence will prove to be irreversible, which has been proposed recently to be a defining feature of senescence *in vivo* [18] remains to be seen, and experiments are ongoing to rigorously test this. Given that ING1a can rapidly and synchronously induce senescence in fibroblasts and our preliminary results suggest that similar effects may be seen in multiple types of cells with characteristics of primary cells, it should prove useful in defining the temporal series of events that occur in these cell types that are responsible for establishing and enforcing a state of cellular senescence through chromatin modification.

## MATERIALS AND METHODS

### Cell culture

Hs68 fibroblast cell strains were obtained from ATCC and were maintained in DMEM (Lonza) supplemented with 10% FBS (Invitrogen) at 37^ο^ C under 5% CO_2_. Young, replication competent cells were of low passages and corresponded to mean population doublings (MPD) between 10 and 25. These strains reached replicative senescence (RS) when they are passaged to MPDs 80 to 85 under our standard tissue culturing conditions [15]. The EA.hy926 cells were a gift from Dr. Andrew Braun and were maintained in DMEM with growth supplements. HaCaT keratinocytes were maintained in high glucose DMEM supplemented with 10% FBS. Both the HaCaT keratinocyte cell line and EAhy926 endothelial cells have many primary cell characteristics, but it should be noted that both are immortalized and somewhat aneuploid. All cells were tested regularly for the presence of mycoplasma using a PCR-based method.

### SIPS treatments

Young fibroblasts were exposed to 70 μM of t-butyl-hydroperoxide that was freshly diluted in growth medium. The cells were exposed to this concentration of t-BHP for 1 hour every day for 7-8 days. After 1 hour exposure time, the cells were washed once with PBS and are allowed to recover in complete growth medium for 24 hours. Doxorubicin induced senescence was by treatment with 100ng/ml of drug for 6-7 days. Adenoviral constructs encoding ING1a and Ras v12 were used to induce senescence by over-expressing ING1a and Ras v12 respectively. Adeno-GFP served as a control for experiments involving Ras v12 and ING1a and young, non-stressed Hs68 cells grown in complete growth medium with no stress agents serve as a control for all experiments. The treatment time was decided based on the time taken for 80% of the cells to stop proliferation as assayed by BrdU incorporation and SA-β-gal staining. The protocol for these two assays were as described previously [15]. The BrdU labelling was carried out at regular intervals to check the exact number of days required for each of the treatments to permanently cause cells to stop proliferating. The data presented here is the quantification of the cells incorporating BrdU after the indicated time points - ING1a after 2 days of infection with Ad-ING1a, Ras v12 after 11 days post infection with Ad-Ras v12, 9 and 7 days of treatment with t-BHP and Doxorubicin respectively. RS was periodically tested after every 5 mean population doublings (MPDs) starting from MPD 65. Under our conditions, Hs68 cells grow to approximately 80-85 MPDs before they become replicatively senescent. These results were compared to Ad-GFP for Ras v12 and ING1a and to young cells for t-BHP, DOXO, and RS. The values for young and Ad-GFP cells that incorporated BrdU was taken as 100% for easier comparison to their respective treatment groups. The experiment was carried out three independent times and the number of cells that incorporated BrdU in each set were counted.

Exogenous expression of ING1a was carried out using an adenoviral system as described [15], except in Figure 3C, in which the cells were transfected with a pCI vector encoding ING1a cDNA using Lipofectamine LTX. This reagent in Hs68 cells gives transfection efficiencies of 20-30%.

### Immunoblotting, immunostaining and phalloidin staining

Hs68 cells treated with the various agents were lysed in Laemmli sample buffer and boiled at 95°C for 10 mins. Proteins were resolved by SDS-PAGE and then blotted using antibodies following standard procedures for blocking and antibody incubations. α-p21, -p16^INK4a^, -actin were purchased from Santa Cruz Biotechnology; α-Rb from BD-Pharmingen; α-ING1a and -p53 are mouse monoclonals produced in the University of Calgary Antibody Facility.

For EGFR internalization, cells grown on coverslips were exposed to various treatments and were serum started overnight. The cells were then stimulated with Texas-Red conjugated EGF (Invitrogen) for 4 mins and then fixed immediately with 4% formaldehyde. The cells were then mounted and imaged using confocal microscopy (Zeiss).

For checking the localization of ING1a with SAHF, cells transfected with pCI-ING1a construct was fixed with 4% formaldehyde, permeabilized with 0.1% Triton X-100, blocked with 5% BSA and were incubated with antibodies against ING1a (red) and HP1γ (green). Nuclei were stained blue using DAPI. The localization of ING1a on the dense DAPI stained regions, which also stain with HP1γ, was analyzed using epifluorescence microscope for visualizing the transfected cells (Axiovision).

For visualizing the actin cytoskeleton, cells exposed to different treatments were fixed with 4% formaldehyde, permeabilized with 0.1% Triton X-100, blocked with 5% BSA and were then incubated with Phalloidin-TRITC (Invitrogen) for 30 mins at room temperature. The cells were then washed thrice with PBS and then mounted for image acquisition.

Expression constructs. Both plasmid and adenoviral ING1a expression constructs will be made freely available. Other constructs used were obtained from sources described previously [15].

## SUPPLEMENTARY MATERIAL FIGURE


