# Supplementary material for: Aging with ING: a comparative study of different forms of stress induced premature senescence
Source: Oncotarget. 2015 Oct 1;6(33):34118–27. doi: 10.18632/oncotarget.5947 (PMC4741440; doi:10.18632/oncotarget.5947)
Supplement: Supplementary file 1 [file oncotarget-06-34118-s001.pdf]

Aging with ING: a comparative study of different forms of stress induced premature senescence

Supplementary Material

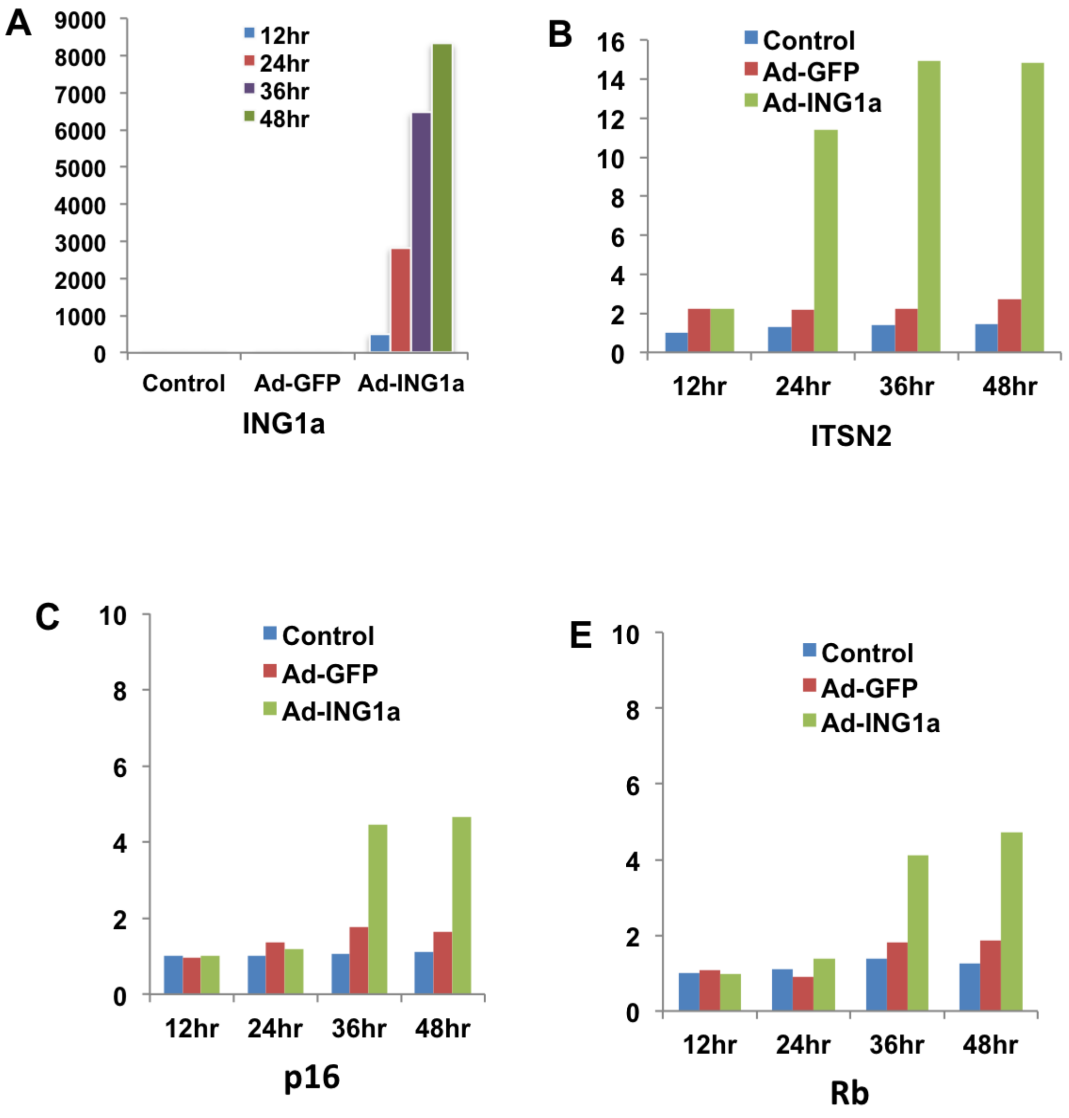

Figure S1. ITSN2 induction precedes the appearance of senescence markers. RNA from uninfected, GFP or ING1a expressing Hs68 cells was isolated at the indicated time points after ING1a expression. Induction of ING1a, ITSN2, $p16^{INK4a}$ and Rb was checked at these time points by qRT-PCR. Figure reproduced from previous publication by Rajarajacholan et al., [15], with permission from PLoS Biology.
